# Nutritional assessment methods for zinc supplementation in prepubertal non-zinc-deficient children

**DOI:** 10.3402/fnr.v59.29733

**Published:** 2015-10-26

**Authors:** Márcia Marília Gomes Dantas Lopes, Naira Josele Neves de Brito, Érika Dantas de Medeiros Rocha, Mardone Cavalcante França, Maria das Graças de Almeida, José Brandão-Neto

**Affiliations:** 1 Postgraduate Program in Health Sciences, Federal University of Rio Grande do Norte, Natal, Brazil; 2 Department of Statistics, Federal University of Rio Grande do Norte, Natal, Brazil; 3 Department of Clinical and Toxicological Analyses, Federal University of Rio Grande do Norte, Natal, Brazil; 4 Department of Internal Medicine, Federal University of Rio Grande do Norte, Natal, Brazil

**Keywords:** zinc supplementation, bioelectrical impedance vector analysis, phase angle, dietary assessment, children

## Abstract

**Background:**

Zinc is an essential nutrient that is required for numerous metabolic functions, and zinc deficiency results in growth retardation, cell-mediated immune dysfunction, and cognitive impairment.

**Objective:**

This study evaluated nutritional assessment methods for zinc supplementation in prepubertal non-zinc-deficient children.

**Design:**

We performed a randomised, controlled, triple-blind study. The children were divided into a control group (10% sorbitol, *n*=31) and an experimental group (10 mg Zn/day, *n*=31) for 3 months. Anthropometric and dietary assessments as well as bioelectrical measurements were performed in all children.

**Results:**

Our study showed (1) an increased body mass index for age and an increased phase angle in the experimental group; (2) a positive correlation between nutritional assessment parameters in both groups; (3) increased soft tissue, and mainly fat-free mass, in the body composition of the experimental group, as determined using bioelectrical impedance vector analysis; (4) increased consumption of all nutrients, including zinc, in the experimental group; and (5) an increased serum zinc concentration in both groups (*p*<0.0001).

**Conclusions:**

Given that a reference for body composition analysis does not exist for intervention studies, longitudinal studies are needed to investigate vector migration during zinc supplementation. These results reinforce the importance of employing multiple techniques to assess the nutritional status of populations.

Optimal growth requires adequate nutrition (1), especially in childhood, when growth and development are rapid. The prevalence of nutritional disorders has increased in children and adolescents in both developed and developing countries (2), and these disorders encompass deficiencies in essential vitamins and minerals (3). Zinc is an essential nutrient that is required for numerous metabolic functions, and its deficiency results in growth retardation, cell-mediated immune dysfunction, and cognitive impairment (4). Although all micronutrient deficiencies are considered as a risk factor for growth delay in children, zinc deficiency is particularly notable (5).

Many nutritional assessment methods, including laboratory, food intake, and anthropometric and body composition assessments, have acceptable accuracy (6). However, methods that can accurately assess anthropometry in children are scarce. It is common to use the body mass index (BMI) to determine nutritional status. International BMI for age cut-offs have been proposed to classify overweight and undernutrition (7, 8), but BMI levels in children should be interpreted with caution. Although a high BMI for age is a good indicator of excess fat mass (FM), differences in BMI among thinner children can be due to fat-free mass (FFM) (9).

Bioelectrical impedance analysis (BIA) is a user-friendly, noninvasive, low-cost, and portable method that can be used to calculate total body fat in children and adults (10). Studies have confirmed that BIA can be a useful tool for assessing body composition (11–13).

Bioelectrical impedance vector analysis (BIVA) visualises impedance measurements (resistance and reactance), and, based on these measurements, body composition can be interpreted. BIVA may be useful for clinical purposes because of its ability to detect changes in hydration or body composition in children (14), specifically in chronic conditions (e.g. chronic obstructive pulmonary disease, anorexia nervosa, cancer, and chronic renal failure) (15–22).

The phase angle is a parameter that is obtained from BIA. This parameter is the deviation of a current that occurs when part of the current is stored across cell membranes, which creates a phase shift that is quantified geometrically as the angular transformation of the reactance–resistance ratio (23, 24). Phase angle measurement could be an effective tool for evaluating clinical outcomes or for monitoring disease progression (25).

Valid and reliable assessment methods for evaluating food intake are critical for identification of the impact of dietary interventions on children's dietary habits and on their health and weight status, and for the future development of successful prevention and intervention strategies (26).

The present study evaluated nutritional assessment methods for zinc supplementation in prepubertal non-zinc-deficient children using anthropometric and bioelectrical measurements as well as dietary assessments.

## Methods

### Subjects

The participants included 62 eutrophic prepubertal children of both sexes who were between 8 and 9 years old; these participants were recruited from three municipal schools in the city of Natal, Brazil. Informed consent was obtained from all subjects and their parents or guardians before participation in the study. The sample was formed using non-probability sampling (convenience sampling). The University Hospital Research Ethics Committee of the Federal University of Rio Grande do Norte (UFRN) approved the protocol (no. 542/11). The Universal Trial Number (UTN) is U1111-1169-3076.

### Inclusion and exclusion criteria

The children had to be eutrophic; healthy; and in Tanner stage 1 for genital, breast, and pubic hair growth. The exclusion criteria included the following: early pubarche, thelarche, or menarche; acute, infectious, or inflammatory disease or a history of disease (neoplasia; diabetes mellitus; nutritional disorders; and liver, kidney, or thyroid disorders); surgery; use of vitamin or mineral supplements; and sex-specific BMI for age, weight for age, and height for age indices outside the interval of −2 to 2 plus *Z*-scores according to 2006 World Health Organization (WHO) curves (27).

### Experimental design

**Fig. 1:**
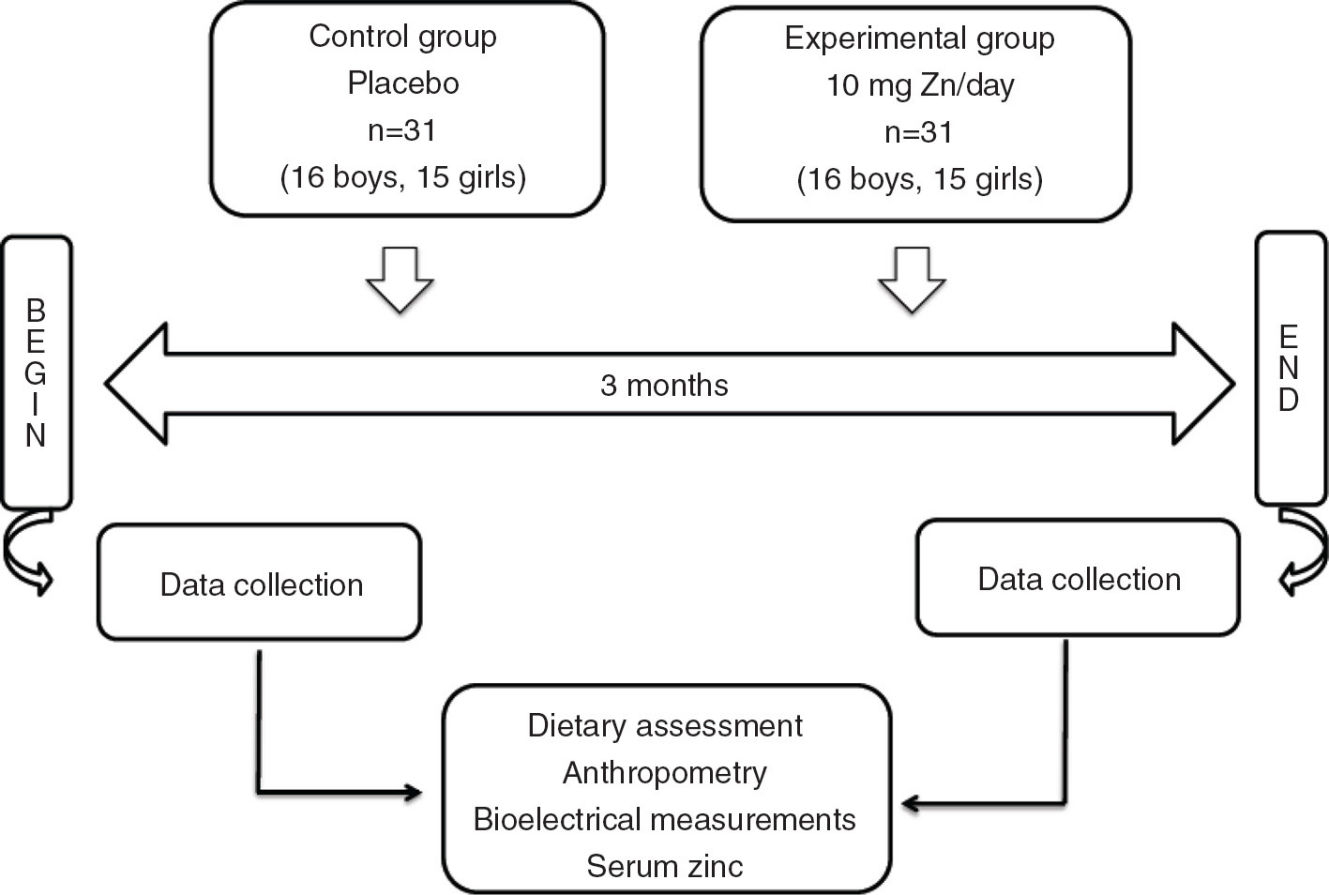
Design of the study.

This study was a randomised, controlled, triple-blind study, and the subjects were divided into two groups: placebo and experimental. These groups, which were supplemented with placebo (10% sorbitol) or 10 mg Zn/day for 3 months, were called the control group and the experimental group, respectively. The children were submitted to anthropometric and bioelectrical measurements and dietary assessments as well as blood collection before and after placebo or oral zinc supplementation. Figure 1 shows the study design. Baseline blood samples were collected from the children at 8 am after a 12-h overnight fast, and on the collection days, the placebo or zinc solution was not administered orally. The patients were in the supine position throughout the test. An antecubital vein was punctured, and venous patency was maintained with a sterile, metal-free saline solution. Venipuncture was performed using plastic, metal-free syringes without a tourniquet.

Zinc vials and zinc intake were monitored every 2 weeks by the same nutritionists during home visits. These nutritionists performed anthropometric measurements and nutritional assessments.

### Anthropometry

Anthropometric measurements were performed after an overnight fast. Weight (in kilograms) and height (in metres) were measured using an electronic balance (Balmak, BK50F, São Paulo, SP, Brazil) and a stadiometer (Stadiometer Professional Sanny, American Medical do Brasil, São Paulo, SP, Brazil), respectively, and BMI was calculated as the ratio of body weight in kilograms to the squared height in metres (kg/m^2^) using the AnthroPlus version 1.0.4 program (www.who.int/growthref/en/). For weight measurements, the child stood on the scale wearing light clothing without shoes. To measure height, the child stood without shoes and with free head props, heels together, arms extended along the body, and the body as upright as possible. The heels, buttocks, shoulders, and head touched the wall or the vertical surface of the measuring equipment. All measurements were performed by the same examiner (28). Growth curves from the WHO for children from 5 to 19 years old were used to obtain classifications of malnutrition, eutrophic, and obesity (29).

Skinfold thicknesses were measured for the triceps (on the back side of the arm, between the olecranon and the acromion) and subscapularis (measured obliquely downward and laterally to the angle of the scapula) using a Lange skinfold calliper (Lange Skinfold Caliper, Beta Technology Incorporated, Cambridge, MD) according to the protocol of Frisancho (30). Parameters for classification established by Heyward and Stolarczyk were used (31). We also measured the circumference of the arm using an inelastic tape measure while the left arm was relaxed, specifically measuring the midpoint between the acromion and the olecranon. From the arm circumference and triceps skinfold thickness data, the arm muscle circumference and arm muscle area were calculated. These parameters were classified according to the percentiles proposed by Frisancho (30), and the FM was calculated using the equation described by Slaughter et al. (32). We evaluated the characteristics of adiposity using the triceps and subscapular skinfold thicknesses and correlated them with the FM obtained by BIA.

### Bioelectrical measurements

After the child had emptied his or her bladder, BIA was performed using a bioelectrical impedance analyser (model Quantum II, RJL Systems, Clinton Township, MI), which requires the precise placement of four surface electrodes (standard tetrapolar placement on the right hand and foot), as reported by Lukaski et al. (33). A painless, low-intensity current of 800 µA at a fixed frequency of 50 kHz was introduced through a surface electrode to the source electrode after the command issued by the device to capture the voltage drop detected by the source electrode was registered under the closest ohm value. There are two components of the whole-body impedance vector: resistance (R), or the opposition to the flow of an alternating current through intra- and extracellular ionic solutions, and reactance (Xc), or the capacitance produced by tissue interfaces and cell membranes across tissues. Both components were recorded from single representative stable measurements by the same operator. We used the BIA equation described by Houtkooper et al. (34). We standardised the BIA measurements using the height (H) of the subjects to eliminate the influence of the conductor length, so both R/H and Xc/H were expressed in ohms per metre (ohm/m).

The impedance analyser (RXc graph)–derived BIVA was performed based on resistance and reactance. Impedance measurements were normalised to the children's height and plotted as bivariate vectors with confidence intervals, which are ellipses in the RXc plane. According to the method proposed by Piccoli et al. (35), when the ellipses of 95% of two mean impedance vectors do not show an overlapping area, they are significantly different (*p*<0.05) in the plane, which is equivalent to a statistically significant result for Hotelling's *T*^2^ test. Otherwise, two mean vectors with overlapping ellipses are equal to a non-significant result for the *T*^2^ test, meaning that these vectors are not different. According to the RXc graph method, we normalised R and Xc measurements to the stature of the subjects (i.e. R/height (H) and Xc/H, expressed in ohm/m), and individual vectors were plotted as points on the RXc graph. The points were subsequently joined to form three tolerance ellipses, which corresponded to the 50th, 75th, and 95th percentiles for the impedance vector distributions of our reference population. To determine the reference bivariate intervals of the whole-body impedance vector in the study groups, the impedance vector was measured by BIA in 62 eutrophic children. The measured R/H and Xc/H values in a group of children were plotted as a mean impedance vector with a 95% confidence ellipsis, and the mean vector position and variability in the corresponding population could be directly established. The reference sample reflects the zinc supplementation distribution among the groups of children from which it was drawn. The graph of the mean RXc was obtained by plotting the 95% confidence ellipses of the mean vectors (i.e. the means of components R/H and Xc/H) for the control and experimental groups. Separated 95% confidence ellipses indicated a significant difference (*p*<0.05) in the vector position (35). BIVA of the RXc method provides more information than the analysis of only the phase angle, which was previously used as an indicator of clinical outcomes and in clinical research studies aimed at identifying disorders in body composition, with good results (36, 37).

The phase angle is derived from two segments of body composition and is calculated using the following formula: PA=arctangent (Xc/R)×180/3.416, as proposed by Barbosa-Silva et al. (38). The phase angle reflects the relative contributions of fluids (R) and cell membranes (Xc) in the human body and is directly proportional to the capacitance and inversely proportional to the resistance (39). Low phase angle values suggest cell death or decreased cell integrity, whereas higher phase angle values suggest a large amount of intact cell membranes (24).

### Dietary assessment

Three-day food records (2 weekdays and 1 weekend day) were completed for all children participating in the study and were assumed to be sufficient for estimating energy and protein intake on an individual basis (40). Each mother was instructed on how to properly describe food and how to observe the food intake and portion sizes of the child. The intake of energy, carbohydrate, protein, fat, fibre, calcium, iron, and zinc was calculated with NutWin software version 1.5 (41). Foods not included in the software were inserted based on food chemical composition tables or nutritional information on the foods’ labels. Additionally, energy, carbohydrate, protein, fat, fibre, calcium, iron, and zinc were analysed according to the recommendations for age and sex (42–45).

The prevalence of inadequate nutrients was calculated according to the estimated average requirement (EAR), considering the potential risk of macronutrient and micronutrient deficiencies.

### Oral zinc administration

The experimental group was supplemented with 10 mg Zn/day (as elemental zinc) in the form of zinc sulphate heptahydrate (ZnSO_4_·7H_2_O; Merck, Darmstadt, Germany)for 3 months. An oral zinc solution was prepared at the Pharmacotechnical Laboratory of the Department of Pharmacy, UFRN. Each drop contained 1 mg of elemental zinc. The control group received an oral placebo under the same conditions. Each group received 10 diluted drops of the syrup added to milk or juice every morning at breakfast. Zinc supplementation was controlled every 2 weeks by the same observer.

### Materials

Vacuette Z Serum Clot Activator Tubes (Greiner Bio-One, Monroe, NC) were used for biochemical analyses. Becton Dickinson (BD) Trace Element Tubes (for serum; BD, Franklin Lakes, NJ) were used for zinc analyses. Polypropylene plastic syringes were purchased from BD, and metal-free plastic tips and tubes were purchased from Bio-Rad Laboratories (Hercules, CA).

### Chemicals

Zinc sulphate heptahydrate (ZnSO_4_·7H_2_O) and Titrisol zinc standard were purchased from Merck.

### Laboratory procedures

All blood samples were collected in the appropriate tubes, and the procedures related to the handling of the zinc samples were performed in accordance with international standards (46). After sample collection, laboratory procedures were performed at the Multidisciplinary Laboratory of Chronic Degenerative Diseases. The blood samples were placed in trace-metal-free tubes without anticoagulants and kept in a stainless steel incubator (FANEM 502, São Paulo, SP, Brazil) for 120 min, until clot formation. Next, 500 µL of serum was collected with a plastic, trace-metal-free pipette and transferred to a plastic tube containing ultrapure water (Milli-Q Plus, Millipore, Billerica, MA) with a final concentration of 2,000 µL to dilute the serum (1:4) for zinc analyses. The samples were kept at −80 °C until subsequent analysis (Ultra-low Freezer, NuAire, Plymouth, MN). Serum zinc samples were analysed in triplicate within the same assay using atomic absorption spectrophotometry (SpectrAA-240FS, Varian, Mulgrave, Victoria, Australia) according to the manufacturer's instructions. The zinc sensitivity was 0.01 µg/mL, the intra-assay coefficient of variation was 2.37%, and the normal reference range was 0.7–1.2 µg/mL. The standard zinc solution (1,000 mg/mL) was obtained by diluting Titrisol zinc standard in ultrapure water. The wavelength used was 213.9 nm, and the lamp current was 10 mA. All other procedures, such as calibrations and measurements, were performed in accordance with the manufacturer's instructions.

### Statistical analyses

Statistical analyses included the D'Agostino and Pearson omnibus normality test, which was used to analyse the normality of all study data. Paired and unpaired Student's *t*-tests were used to compare the data obtained within the control and experimental groups or between the groups. The Wilcoxon matched-pairs signed-rank test was used to complement the paired nonparametric test, and the Mann–Whitney test was used to complement the unpaired nonparametric test. The correlation between two variables within each subject was determined using Spearman's coefficient for nonparametric correlation. Statistical tests were performed using GraphPad Prism version 6.0 software (GraphPad Software, Inc., San Diego, CA). The statistical analyses of the BIVA were performed using Microsoft Excel 2003 (BIVA software) (39). All comparisons were considered statistically significant at the 5% significance level.

## Results

### Study group

In total, 62 schoolchildren were examined. This study specifically included 32 boys and 30 girls, who were all submitted to anthropometric and dietary assessments and blood collection (Fig. 1). The sample size of 62 schoolchildren was adequate for this study because for any δ value ≥−0.09, the sample size required would be *n*=15.

### Anthropometry

According to BMI for age classification, all schoolchildren were eutrophic during the 3-month study. At the end of the study, BMI for age was significantly different for the experimental group (Table 1).

**Table 1:** Anthropometry and body composition before and after placebo (control group) or oral zinc supplementation (experimental group) in 62 prepubertal children between 8 and 9 years old of both sexes

Parameter	Water free (%)	*p*	Fat-free mass (%)	*p*	Fat mass (%)	*p*	BMI for age (kg/m^2^)	*p*	Phase angle (φ)	*p*
CG-Before	59.26±3.28	0.0305*	81.13±4.35	0.0080*	18.87±4.35	0.0080*	15.83±1.55	0.0563	5.31±0.64	0.1582
CG-After	58.61±3.40		80.03±4.64		19.97±4.80		16.02±1.80		5.34±0.56	
EG-Before	59.23±3.84	0.4979	81.00±5.29	0.3831	19.00±5.29	0.3831	15.92±1.76	0.0005*	5.27±0.58	0.0470*
EG-After	59.10±3.50		80.74±4.85		19.26±4.85		16.33±2.03		5.39±0.65	
CG-Before	59.26±3.28	0.7659	81.13±4.35	0.7231	18.87±4.35	0.7231	15.83±1.55	0.8455	5.31±0.64	0.9327
EG-Before	59.23±3.84		81.00±5.29		19.00±5.29		15.92±1.76		5.27±0.58	
CG-Before	59.26±3.28	0.8520	81.13±4.35	0.7419	18.87±4.35	0.7419	15.83±1.55	0.4856	5.31±0.64	0.4728
EG-After	59.10±3.50		80.74±4.85		19.26±4.85		16.33±2.03		5.39±0.65	
CG-After	58.61±3.40	0.3109	80.03±4.64	0.2166	19.97±4.80	0.2166	16.02±1.80	0.8230	5.34±0.56	0.6469
EG-Before	59.23±3.84		81.00±5.29		19.00±5.29		15.92±1.76		5.27±0.58	
CG-After	58.61±3.40	0.5829	80.03±4.64	0.5582	19.97±4.80	0.5582	16.02±1.80	0.6023	5.34±0.56	0.7227
EG-After	59.10±3.50		80.74±4.85		19.26±4.85		16.33±2.03		5.39±0.65	

Values are expressed as the means±SD

* *p*<0.05.

Paired Student's *t*-tests were used to compare the data obtained within the control and experimental groups. Unpaired Student’ t-tests was used to compare the data obtained between both control and experimental groups. Control group before (CG-Before) and control group after (CG-After) represent before and after oral placebo administration, respectively. Experimental group before (EG-Before) and experimental group after (EG-After) represent before and after oral zinc supplementation, respectively.

### Correlation between %FM determined by skinfold thickness and %FM determined by BIA

**Fig. 2:**
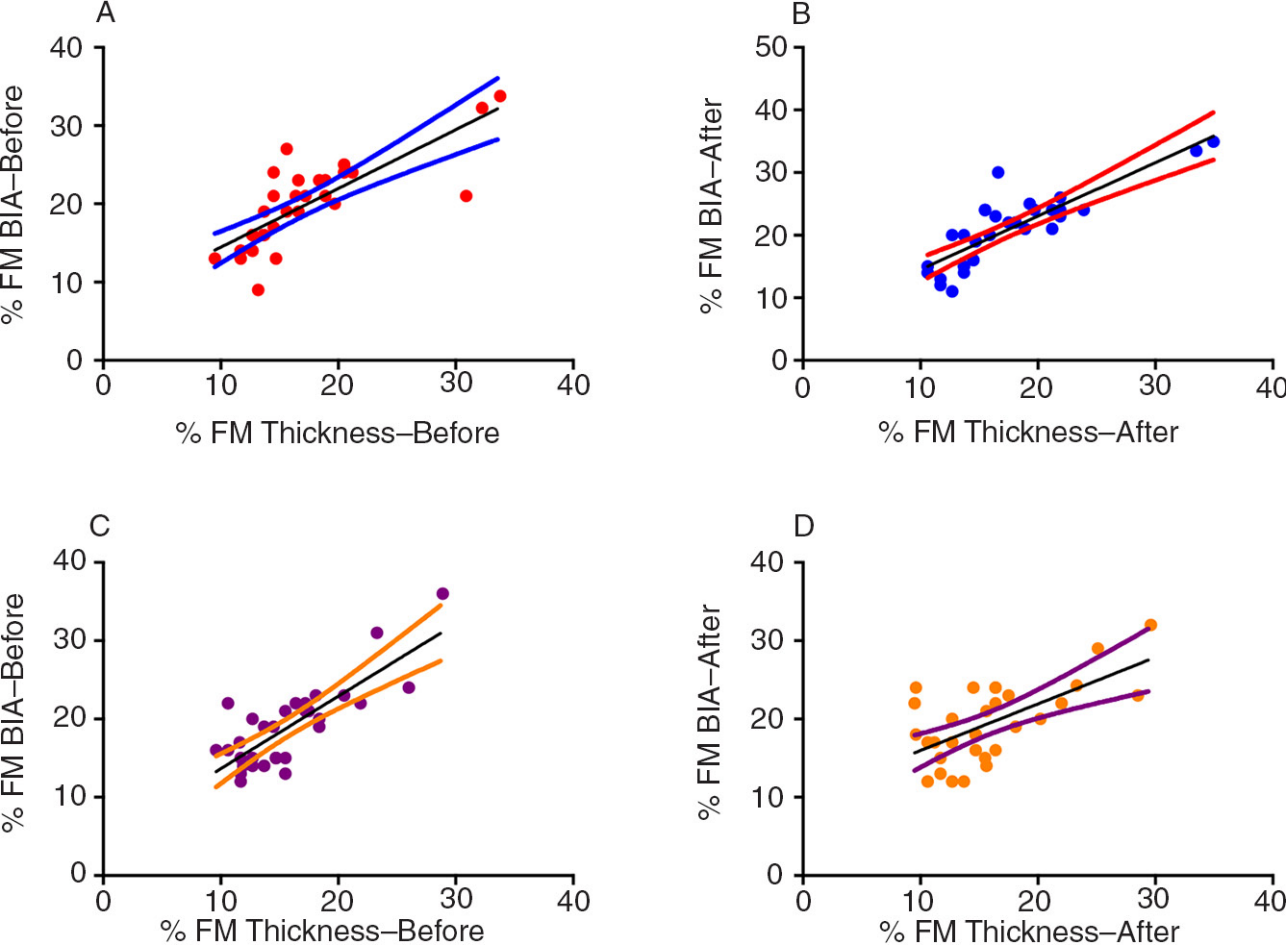
Values of correlation between (a) %FM by BIA and %FM by skinfold thickness in the control group before placebo (*r*=0.8218, *p*<0.0001), (b) %FM by BIA and %FM by skinfold thickness in the control group after placebo (*r*=0.8449, *p*<0.0001), (c) %FM by BIA and %FM by skinfold thickness in the experimental group before zinc supplementation (*r*=0.6639, *p*<0.0001), and (d) %FM by BIA and %FM by skinfold thickness in the experimental group after zinc supplementation (*r*=0.5159, *p*=0.0030).

In our study, we found a strong positive correlation between FM measured based on the skinfold thickness and FM measured by BIA in the control group and experimental group (Fig. 2).

### Bioelectrical measurements

There were changes in the body composition of the control group, with increased FM and decreased FFM and water free, whereas there was no difference in these parameters after supplementation in the experimental group (Table 1). A significant increase in the phase angle was observed after supplementation, which most likely indicated a higher cell mass in the body (Table 1).

**Fig. 3:**
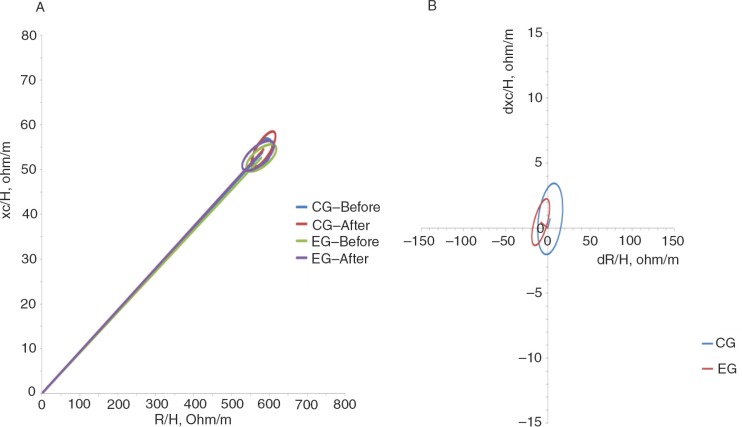
Confidence ellipses for mean vectors. (a) Mean impedance vectors and (b) paired graphs with the 95% confidence ellipses for healthy, eutrophic children between 8 and 9 years of age before and after placebo administration in the control group and oral zinc supplementation in the experimental group.

Assuming a bivariate normal distribution for R/H and Xc/H, we calculated the bivariate 95% confidence limits for the mean impedance vectors of the different classification groups (i.e. the ellipsis within which the two-dimensional mean vectors fall with a 95% probability). We called the average of R/H and Xc/H recorded in the groups of children the ‘RXc mean graph’ (35). The mean vectors for the control group and experimental group before and after treatment were plotted and compared using the unpaired two-sample Hotelling's *T*^2^ test (Fig. 3a). The 95% confidence ellipses of the two mean vectors of each group were overlapping, which means that the positions between the vectors from the control group and experimental group were not significantly different in the RXc plane. The paired one-sample Hotelling's *T*^2^ test determined a difference in mean vectors between the first measurement (before supplementation) and the matched second measurement (after supplementation). The experimental group showed an increase in soft tissue, and mainly FFM, in the body composition analysis after oral zinc supplementation compared with before supplementation (*p*<0.0001) (Fig. 3b).

### Dietary assessment

Energy, carbohydrate, protein, fat, iron, and zinc levels were normal according to the recommendations by age and sex, and these values showed a significant increase after oral zinc supplementation, which indicated increased food consumption after the 3-month study (*p*<0.0001). Additionally, fibre and calcium concentrations increased after oral zinc supplementation, although they remained low compared with the recommendations by age and sex (Table 2).

**Table 2:** Energy and nutrient intake before and after placebo (control group) or oral zinc supplementation (experimental group) compared with their recommendations by age and sex

Parameter	EAR	Control group before	Control group after	Experimental group before	Experimental group after
Energy (kcal/day)	6–9 years (boys): 1,573–1,978 kcal/day [42]	1,544±165.70	1,552±170.80	1,655±212.80	1,831±233.50
	6–9 years (girls): 1,428–1,854 kcal/day [42]	*p=0.0313		*p<0.0001	
			*p<0.0001		
Carbohydrate (g/day)	100 g/day [44]	181.00±10.12	182.10±10.11	181.90±17.09	184.50±17.09
		*p<0.0001		*p<0.0001	
			*p<0.0187		
Protein (g/kg/day)	4–8 years (both sexes): 0.76 g/kg/day [44]	39.76±2.32	40.13±2.42	41.58±3.32	47.22±3.37
	9–13 years (both sexes): 0.76 g/kg/day [44]	*p=0.0014		*p<0.0001	
			*p<0.0001		
Fat (g/day)	ND [44]	35.72±2.14	35.91±2.15	36.16±1.90	39.88±2.77
		*p<0.0010		*p<0.0001	
			*p<0.0001		
Fibre (g/day)	4–8 years (both sexes): 25 g/day [44]	10.66±1.14	11.09±1.11	10.17±1.07	11.59±1.06
	9–13 years (girls): 26 g/day [44]	*p<0.0001		*p<0.0001	
	9–13 years (boys): 31 g/day [44]		*p*=0.0873		
Calcium (mg/day)	4–8 years (both sexes): 800 mg/day [45]	627.40±103.80	622.70±89.93	582.60±64.34	638.70±61.15
	9–13 years (both sexes): 1,100 mg/day [45]	*p*=0.4728		*p<0.0001	
			*p=0.0422		
Iron (mg/day)	4–8 years (both sexes): 4.1 mg/day [43]	8.88±0.73	9.08±0.68	8.73±0.45	9.31±0.42
	9–13 years (boys): 5.9 mg/day [43]	*p=0.0004		*p<0.0001	
	9–13 years (girls): 5.7 mg/day [43]		*p*=0.1174		

Values are expressed as the means±SD

* *p*<0.05.

Paired Student's *t*-tests were used to compare the data obtained within the control and experimental groups. Unpaired Student's *t*-tests were used to compare the data obtained between the control and experimental groups. Control group before and control group after represent before and after oral placebo, respectively. Experimental group before and experimental group after represent before and after oral zinc supplementation, respectively.

**Fig. 4:**
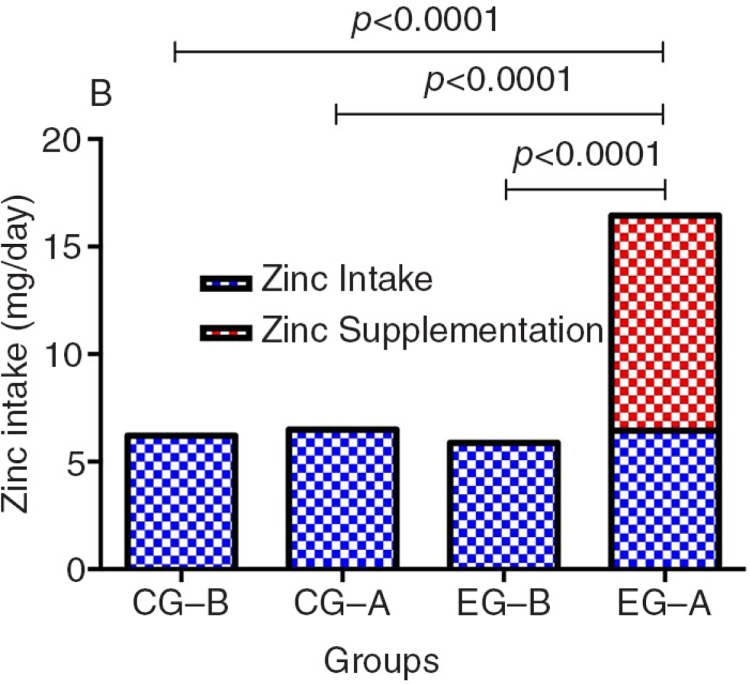
Estimated average requirement for zinc in both sexes: 4–8 years = 4 mg/day and 9–13 years= 7 mg/day (43). Different comparisons were conducted using a paired Student's *t*-test in the control group and the experimental group and an unpaired Student's *t*-test comparing the experimental group versus the control group. CG-B means control group before oral placebo administration. CG-A means control group after oral placebo administration. EG-B means experimental group before oral zinc supplementation. EG-A means experimental group after oral zinc supplementation. Values are expressed as the mean.

After supplementation, zinc intake plus zinc supplementation did not exceed the upper limit (UL) of this nutrient (Fig. 4).

### Oral zinc administration

There were increases in the serum zinc concentration in the control group from before (0.9369±0.1297 µg/mL) to after (1.0610±0.1251 µg/mL) placebo administration and in the experimental group from before (0.8934±0.1244 µg/mL) to after (1.0750±0.1113 µg/mL) zinc supplementation. Significant differences were observed between the control group before and after placebo administration (*p*<0.0001) and in the experimental group before and after zinc supplementation (*p*<0.0001).

## Discussion

### Study group

Among the 62 children evaluated, we examined the effect of zinc supplementation on nutritional status using anthropometry, conventional BIA, BIVA, and analyses of the phase angle and food intake.

### Anthropometry

In a previous study conducted by our team, we suggested that an oral supplementation dose of 5 mg Zn/day significantly increased height (47). The children in the present study, who ingested 10 mg Zn/day, exhibited the same clinical characteristics, and they were followed using the same methodological design as in the previous investigation. In another study that evaluated the effect of zinc supplementation on growth by using 10 mg Zn/day for 4 months, higher levels of serum zinc were found in the supplemented group, and weight and height development was observed in both supplemented and non-supplemented groups (48). Additionally, zinc supplementation with 5 mg Zn/day for a mean period of 4 months in full-term, normal infants had a significant effect on the skinfold thickness, but it had no effect on the infants’ linear growth (49).

### Bioelectrical measurements

Knowledge regarding the values of the phase angle in healthy populations allows improved utilisation in clinical practice (50–52). Typical phase angle values in children aged 10 years are 6.2 for girls and 6.3 for boys, and these values increase with an increase in weight, BMI, FFM, and total body weight (TBW) (53). There are no data on the phase angle after zinc supplementation in the literature. However, the phase angle was used in a study of obese patients; patients undergoing chronic haemodialysis; and patients with cirrhosis, cancer, or human immunodeficiency virus (HIV) infection, and the results confirmed its role as an important marker of mortality and morbidity (13, 38, 54).

The increase in the FM and the decrease in the lean body mass and TBW of the control group may be justified by dietary habits and the socioeconomic conditions of this population. For instance, in Brazilian studies of children of both sexes under 10 years old, there is inadequate consumption of fruits and greens. Additionally, sweet drinks account for a large proportion of total energy and carbohydrate consumption, and candies and sweets account for a large proportion of the total consumption of lipids in particular (55, 56). Moreover, we found a positive correlation between the %FM measured by BIA and the %FM measured based on the skinfold thickness in the control and experimental groups. Similar results were found by Pecorato et al. (57), who also reported that BIA may be a more precise method than skinfold thickness assessment for measuring the %FM in studies in the paediatric population.

BIVA was used to observe whether there were any changes in body composition with oral zinc supplementation. BIVA patterns are based exclusively on the electrical properties of tissues, without considering information about body weight. BIVA can monitor changes in both tissue hydration (the R component) and structure (the Xc component) because both components of the impedance vector are considered simultaneously. Studies have shown that the advantage of BIVA is that it is a good identifier of individual vectors, indicating alterations in tissue hydration and body structure in subjects from any BMI class (14). BIVA should be considered as an assessment and monitoring tool (unpublished results).

### Dietary assessment

All of the children were eutrophic during the 3-month study presented here, and there was increased food consumption in the experimental group, as indicated by significant increases in energy, carbohydrate, protein, fat, fibre, calcium, iron, and zinc levels after oral zinc supplementation compared with control treatment. This effect can be attributed to the fact that oral zinc supplementation increased the children's appetite, as noted among all children in the experimental group.

Assessment of dietary intake has been shown to be an essential factor for understanding the relationship between diet and health, particularly in relation to the intake of certain micronutrients and nutritional status. Additionally, adequate consumption of micronutrients is essential for the maintenance of metabolic functions (58). Studies have shown that zinc supplementation improves the acceptance of salty foods by children and adolescents (59, 60).

### Oral zinc administration

The baseline serum zinc concentrations increased significantly after 5 mg Zn/day supplementation for 3 months in children aged 6–9 years (61). Serum zinc concentrations were reported to significantly increase only in the experimental group in a study by Rocha et al. (62). Another study revealed an increase in baseline serum zinc concentrations in the control group but a more significant increase in the experimental group (personal communication). Our results are likely due to the different sample size and also to the different feeding patterns of the children studied. The tolerable upper zinc intake level and the baseline serum zinc concentrations remained in the normal reference ranges, and no side effects were observed at a dose of 10 mg Zn/day.

### Limitations

One major limitation of the study is the relatively small sample size. However, this limitation was circumvented by using a non-probabilistic sample. Moreover, calculation of the sample of 62 children showed that the results are statistically reliable.

## Conclusions

In conclusion, our results showed (1) an increased BMI for age and an increased phase angle in the experimental group; (2) a positive correlation between nutritional assessment parameters in both groups; (3) increased soft tissue, and mainly FFM, in the body composition of the experimental group, as determined using BIVA; and (4) increased consumption of all nutrients in the experimental group. Because a reference for BIVA does not exist for intervention studies, longitudinal data are needed to investigate vector migration during zinc supplementation. The reference values of the impedance vector can be used for clinical practice to assess children's body composition. Additionally, few studies have compared different methods, and no nutritional assessment method alone is good to evaluate the nutritional status of populations. In this sense, our results reinforce the importance of employing multiple techniques, such as anthropometric methods, BIA, BIVA, phase angle analysis, and dietary assessment, in the evaluation of the nutritional status of non-zinc-deficient children during oral zinc supplementation.

## References

[1] Sozeri B, Mir S, Kara OD, Dincel N (2011). Growth impairment and nutritional status in children with chronic kidney disease. Iran J Pediatr.

[2] Ogden CL, Carroll MD (2010). Prevalence of obesity among children and adolescents: United States, trends 1963–1965 through 2007–2008. Health E-Stat.

[3] Black RE, Allen LH, Bhutta ZA, Caulfield LE, de Onis M, Ezzati M (2008). Maternal and Child Undernutrition Study Group. Maternal and child undernutrition: global and regional exposures and health consequences. Lancet.

[4] Prasad AS (2013). Discovery of human zinc deficiency: its impact on human health and disease. Adv Nutr.

[5] Rivera JA, Hotz C, González-Cossío T, Neufeld L, García-Guerra A (2003). The effect of micronutrient deficiencies on child growth: a review of results from community-based supplementation trials. J Nutr.

[6] Wells JC, Fewtrell MS (2008). Is body composition important for paediatricians?. Arch Dis Child.

[7] Cole TJ, Bellizzi MC, Flegal KM, Dietz WH (2000). Establishing a standard definition for child overweight and obesity worldwide: international survey. BMJ.

[8] Cole TJ, Flegal KM, Nicholls D, Jackson AA (2007). Body mass index cut offs to define thinness in children and adolescents: international survey. BMJ.

[9] Freedman DS, Wang J, Maynard LM, Thornton JC, Mei Z, Pierson RN (2005). Relation of BMI to fat and fat-free mass among children and adolescents. Int J Obes (Lond).

[10] L'Abée C, Poorts-Borger PH, Gorter EH, Piccoli A, Stolk RP, Sauer PJ (2010). The bioelectrical impedance vector migration in healthy infants. Clin Nutr.

[11] Savino F, Grasso G, Cresi F, Oggero R, Silvestro L (2003). Bioelectrical impedance vector distribution in the first year of life. Nutrition.

[12] Kushner RF, Gudivaka R, Schoeller DA (1996). Clinical characteristics influencing bioelectrical impedance analysis measurements. Am J Clin Nutr.

[13] Kyle UG, Bosaeus I, De Lorenzo AD, Deurenberg P, Elia M, Manuel Gómez J (2004). Bioelectrical impedance analysis-part II: utilization in clinical practice. Clin Nutr.

[14] Guida B, Pietrobelli A, Trio R, Laccetti R, Falconi C, Perrino NR (2008). Body mass index and bioelectrical vector distribution in 8-year-old children. Nutr Metab Cardiovasc Dis.

[15] Chertow GM, Lazarus JM, Lew NL, Ma L, Lowrie EG (1997). Bioimpedance norms for the hemodialysis population. Kidney Int.

[16] Piccoli A (1998). Identification of operational clues to dry weight prescription in hemodialysis using bioimpedance vector analysis. The Italian hemodialysis-Bioelectrical Impedance Analysis (HD-BIA) Study Group. Kidney Int.

[17] Guida B, De Nicola L, Trio R, Pecoraro P, Iodice C, Memoli B (2000). Comparison of vector and conventional bioelectrical impedance analysis in the optimal dry weight prescription in hemodialysis. Am J Nephrol.

[18] Pillon L, Piccoli A, Lowrie EG, Lazarus JM, Chertow GM (2004). Vector length as a proxy for the adequacy of ultrafiltration in hemodialysis. Kidney Int.

[19] Walter-Kroker A, Kroker A, Matticucci-Guehlke M, Glaab T (2011). A practical guide to bioelectrical impedance analysis using the example of chronic obstructive pulmonary disease. Nutr J.

[20] Piccoli A, Codognotto M, Di Pascoli L, Boffo G, Caregaro L (2005). Body mass index and agreement between bioimpedance and anthropometry estimates of body compartments in anorexia nervosa. JPEN J Parenter Enteral Nutr.

[21] Brooks ER, Fatallah-Shaykh SA, Langman CB, Wolf KM, Price HE (2008). Bioelectric impedance predicts total body water, blood pressure, and heart rate during hemodialysis in children and adolescents. J Ren Nutr.

[22] Malecka-Massalska T, Smolen A, Zubrzycki J, Lupa-Zatwarnicka K, Morshed K (2012). Bioimpedance vector pattern in head and neck squamous cell carcinoma. J Physiol Pharmacol.

[23] Selberg O, Selberg D (2002). Norms and correlates of bioimpedance phase angle in healthy human subjects, hospitalized patients, and patients with liver cirrhosis. Eur J Appl Physiol.

[24] Baumgartner RN, Chumlea WC, Roche AF (1988). Bioelectric impedance phase angle and body composition. Am J Clin Nutr.

[25] Barbosa-Silva MC, Barros AJ, Wang J, Heymsfield SB, Pierson RN (2005). Bioelectrical impedance analysis: population reference values for phase angle by age and sex. Am J Clin Nutr.

[26] Biltoft-Jensen A, Hjorth MF, Trolle E, Christensen T, Brockhoff PB, Andersen LF (2013). Comparison of estimated energy intake using Web-based Dietary Assessment Software with accelerometer-determined energy expenditure in children. Food Nutr Res.

[27] WHO, Multicentre Growth Reference Study Group (2006). WHO child growth standards: length/height-for-age, weight-for-age, weight-for-length, weight-for-height and body mass index-for-age: methods and development.

[28] Brasil, Ministério da Saúde Vigilância Alimentar e Nutricional – SISVAN (2004). Orientaç[otilde]es básicas para a coleta, processamento, análise dos dados e informação em serviços de saúde.

[29] de Onis M, Onyango AW, Borghi E, Siyam A, Nishida C, Siekmann J (2007). Development of a WHO growth reference for school-aged children and adolescents. Bull World Health Organ.

[30] Frisancho AR (2008). Anthropometric standards: an interactive nutritional reference of body size and body composition for children and adults.

[31] Heyward VH, Stolarczyk LM (2000). Avaliação da Composição Corporal Aplicada.

[32] Slaughter MH, Lohman TG, Boileau RA, Horswill CA, Stillman RJ, Van Loan MD (1988). Skinfold equations for estimation of body fatness in children and youth. Hum Biol.

[33] Lukaski HC, Bolonchuk WW, Hall CB, Siders WA (1986). Validation of tetrapolar bioelectrical impedance method to assess human body composition. J Appl Physiol.

[34] Houtkooper LB, Going SB, Lohman TG, Roche AF, Van Loan M (1992). Bioelectrical impedance estimation of fat-free mass in children and youth: a cross validation study. J Appl Physiol.

[35] Piccoli A, Rossi B, Pillon L, Bucciante G (1994). A new method for monitoring body fluid variation by bioimpedance analysis: the RXc graph. Kidney Int.

[36] Piccoli A, Pastori G (2002). BIVA software.

[37] Tanabe RF (2010). Valores de referência do vetor de bioimpedância elétrica corporal total em lactentes e pré-escolares [dissertação].

[38] Barbosa-Silva MC, Barros AJ, Post CL, Waitzberg DL, Heymsfield SB (2003). Can bioelectrical impedance analysis identify malnutrition in preoperative nutrition assessment?. Nutrition.

[39] Gupta D, Lis CG, Dahlk SL, Vashi PG, Grutsch JF, Lammersfeld CA (2004). Bioelectrical impedance phase angle as a prognostic indicator in advanced pancreatic cancer. Br J Nutr.

[40] Bingham SA, Nelson M, Paul AA, Haraldsdottir J, Loken EB, Van Staveren WA, Cameron ME, van Starveren WA (1988). Methods for data collection at an individual level. Manual on methodology for food consumption studies.

[41] UNICAMP (2011). Núcleo de Estudos e Pesquisas em Alimentação (NEPA). Tabela Brasileira de Composição de Alimentos (TACO).

[42] Human energy requirements (2001). Report of the Joint FAO/WHO/UNU Expert Consultation.

[43] Institute of Medicine, Food and Nutrition Board (2003). Dietary reference intakes for vitamin A, vitamin K, arsenic, boron, chromium, copper, iodine, iron, manganese, molybdenum, nickel, silicon, vanadium, and zinc.

[44] Institute of Medicine, Food and Nutrition Board (2005). Dietary reference intakes (DRIs) for energy, carbohydrate, fiber, fat, fatty acids, cholesterol, protein, and amino acids.

[45] Institute of Medicine, Food and Nutrition Board (2011). Dietary reference intakes for calcium and vitamin D.

[46] Hess SY, Peerson JM, King JC, Brown KH (2007). Use of serum zinc concentration as an indicator of population zinc status. Food Nutr Bull.

[47] Alves CX, Vale SHL, Dantas MMG, Maia AA, Franca MC, Marchini JS (2012). Positive effects of zinc supplementation on growth, GH, IGF1, and IGFBP3 in eutrophic children. J Pediatr Endocrinol Metab.

[48] Silva APR, Vitolo MR, Zara LF, Castro CFS (2006). Effects of zinc supplementation on 1- to 5-year old children. J Pediatr (Rio J).

[49] Radhakrishna KV, Hemalatha R, Babu Geddam JJ, Kumar AP, Balakrishna N, Shatrugna V (2013). Effectiveness of zinc supplementation to full term normal infants: a community based double blind, randomized, controlled, Clinical Trial. PLoS One.

[50] Meireles MS, Wazlawik E, Bastos JL, Garcia MF (2012). Comparison between nutritional risk tools and parameters derived from bioelectrical impedance analysis with subjective global assessment. J Acad Nutr Diet.

[51] Kyle UG, Genton L, Pichard C (2013). Low phase angle determined by bioelectrical impedance analysis is associated with malnutrition and nutritional risk at hospital admission. Clin Nutr.

[52] Kyle UG, Soundar EP, Genton L, Pichard C (2012). Can phase angle determined by bioelectrical impedance analysis assess nutritional risk? A comparison between healthy and hospitalized subjects. Clin Nutr.

[53] Martirosov EG, Khomyakova IA, Pushkin SV, Romanova TF, Semenov MM, Rudnev SG (2007). Bioelectric impedance phase angle and body composition in Russian children aged 10–16 years: reference values and correlations.

[54] Fernandes SA, Bassani L, Nunes FF, Aydos ME, Alves AV, Marroni CA (2012). Nutritional assessment in patients with cirrhosis. Arq Gastroenterol.

[55] Conceição SIO, Santos CJN, Silva AAM, Silva JS, Oliveira TC (2010). Food consumption of schoolchildren from private and public schools of São Luis, Maranhão, Brazil. Rev Nutr.

[56] Hinnig PF, Bergamaschi DP (2012). Food items in the food intake of children aged seven to ten years. Rev Bras Epidemiol.

[57] Pecoraro P, Guida B, Caroli M, Trio R, Falconi C, Principato S (2003). Body mass index and skinfold thickness versus bioimpedance analysis: fat mass prediction in children. Acta Diabetol.

[58] Leão ALM, dos Santos LC (2012). Micronutrient consumption and overweight: is there a relationship?. Rev Bras Epidemiol.

[59] Campos D, Veras Neto MC, Silva Filho V, Leite MF, Holanda MB, Cunha NF (2004). Zinc supplementation may recover taste for salt meals. J Pediatr (Rio J).

[60] Tupe RP, Chiplonkar SA (2009). Zinc supplementation improved cognitive performance and taste acuity in Indian adolescent girls. J Am Coll Nutr.

[61] de Moura JE, de Moura ENO, Alves CX, Vale SH, Dantas MM, Silva AA (2013). Oral zinc supplementation may improve cognitive function in schoolchildren. Biol Trace Elem Res.

[62] Rocha EDM, Brito NJN, Dantas MMG, Silva AA, Almeida MG, Brandão-Neto J (2015). Effect of zinc supplementation on GH, IGF1, IGFBP3, OCN and ALP in non-zinc-deficient children. J Am Coll Nutr.

